# Rice-Magnaporthe oryzae interactions in resistant and susceptible rice cultivars under panicle blast infection based on defense-related enzyme activities and metabolomics

**DOI:** 10.1371/journal.pone.0299999

**Published:** 2024-03-07

**Authors:** Xiurong Yang, Shuangyong Yan, Guangsheng Li, Yuejiao Li, Junling Li, Zhongqiu Cui, Shuqin Sun, Jianfei Huo, Yue Sun

**Affiliations:** 1 Institute of Plant Protection, Tianjin Academy of Agricultural Sciences, Tianjin, P.R.China; 2 Institute of Crop Research, Tianjin Academy of Agricultural Sciences, Tianjin, P.R.China; Osmania University, INDIA

## Abstract

Rice blast, caused by rice blast fungus (*Magnaporthe oryzae*), is a global threat to food security, with up to 50% yield losses. Panicle blast is a severe form of rice blast, and disease responses vary between cultivars with different genotypes. Reactive oxygen species (ROS)-mediated signaling reactions and the phenylpropanoid pathway are important defense mechanisms involved in recognizing and resisting against fungal infection. To understand rice-*M*. *oryzae* interactions in resistant and susceptible cultivars, we determined dynamic changes in the activities of five defense-related enzymes in resistant cultivar jingsui 18 and susceptible cultivar jinyuan 899 infected with *M*. *oryzae* from 4 to 25 days after infection. We then performed untargeted metabolomics analyses to profile the metabolomes of the cultivars under infected and non-infected conditions. Dynamic changes in the activities of five defense-related enzymes were closely related to panicle blast resistance in rice. Metabolome data analysis identified 634 differentially accumulated metabolites (DAMs) between resistant and susceptible cultivars following infection, potentially explaining differences in disease response between varieties. The most enriched DAMs were associated with lipids and lipid-like molecules, phenylpropanoids and polyketides, organoheterocyclic compounds, organic acids and derivatives, and lignans, neolignans, and related compounds. Multiple metabolic pathways are involved in resistance to panicle blast in rice, including biosynthesis of other secondary metabolites, amino acid metabolism, lipid metabolism, phenylpropanoid biosynthesis, arachidonic acid metabolism, arginine biosynthesis, tyrosine metabolism, tryptophan metabolism, tyrosine and tryptophan biosynthesis, lysine biosynthesis, and oxidative phosphorylation.

## Introduction

Rice blast disease, caused by the fungus (*Magnaporthe oryzae*), has become one of the most destructive diseases in rice-growing regions globally, as it can lead to up to 50% yield losses if ideal conditions are attained [1,2]. Panicle blast is the most damaging type of blast, and responses to infection depend on genotype [3]. Therefore, the identification of disease-resistant genetic resources and selection of varieties with high resistance is an economical and effective approach for disease management. In addition, due to the lack of research surrounding panicle blast in rice, it is key to understand the resistance mechanisms in resistant and susceptible cultivars, in terms of their interaction with *M*. *oryzae*.

A number of studies have uncovered that plants can establish effective defense responses to recognize and resist against fungal infection through the activation of complex defense processes [4,5]. One of these is the generation of reactive oxygen species (ROS) and establishing a balance between ROS production and scavenging during pathogen-plant interactions [6]. ROS scavenging enzymes including superoxide dismutase (SOD), peroxidase (POD), and catalase (CAT) are key components for regulating ROS levels and are also pertinent to plant disease resistance [7]. Furthermore, the phenylpropanoid pathway has been acknowledged to be important in higher plants [8]. Phenylalanine ammonia-lyase (PAL), the initial enzyme in the pathway, is involved in the synthesis of a range of defensive lignin and phenolic compounds and thereby plays a critical role in plant defense systems [9,10]. Polyphenol oxidase (PPO) is also an essential enzyme in producing phenolic compounds that shield against plant pathogens [7]. In addition, ROS and ROS scavenging enzymes have been reported to mediate resistance against *M*. *oryzae* [11]. Rice phenylalanine ammonia-lyase gene *OsPAL4* is linked to broader-spectrum disease resistance, while *OsPAL2* and *OsPAL6* are associated with disease resistance to a variety of pathogens [12]. The activities of defense-related enzymes may be very important for evaluating resistance to panicle blast in rice cultivars with different genotypes. However, information regarding the mechanisms and progression of resistance to panicle blast via antioxidative defense systems and the phenylpropanoid pathway in rice remains scarce.

Metabolites produced by plants are known to play important roles in plant defense responses against both biotic and abiotic stresses [13]. Recent progress in metabolomics has enabled detailed information about biochemical changes occurring at cellular and tissue levels in plants to be obtained and applied in the study of the interactions between plants and pathogens [14–17]. For example, certain metabolites have been identified and employed as resistance biomarkers in a range of plants, including tomatoes [18], soybean [19], grapes [20] and rice [13], infected by various species of phytopathogens. Furthermore, metabolomic data can be leveraged to develop treatments that employ key metabolites to promote plant growth and disease resistance, thus offering an effective approach for protecting farmland from potential hazards [21].

The molecular mechanisms underlying rice blast resistance have been investigated, and many blast resistance genes have been cloned [22,23]. However, the biochemical mechanisms involved in the interaction between rice and the pathogen *M*. *oryzae* remain elusive, limiting the development of improved panicle blast resistance in rice. In order to gain insight into the biochemical mechanisms of panicle blast resistance, this study profiled the metabolome of resistant and susceptible varieties of rice and identified compounds and associated metabolic pathways related to panicle resistance, particularly antioxidative defense systems and the phenylpropanoid pathway. This study is the first to report in detail the biochemical mechanisms associated with resistance to panicle blast in rice, and the findings provide valuable data that can be used in the prevention of blast disease.

## Materials and methods

### Plant materials and growth conditions

In this study, two japonica rice cultivars, panicle blast-resistant cultivar jinsui18 and susceptible cultivar jinyuan899, were utilized. These two cultivars exhibited considerable variations in their resistance to panicle blast, with the same heading date and growth period. Moreover, the plants were cultivated in the netting house at the WuQing Experimental Base in Tianjin, with a row pitch of 20 cm and a row space of 20 cm. Disease resistance tests involving *M*. *oryzae* inoculation were conducted for two years, from 2020 to 2021.

### *M*. *oryzae* strains, culture conditions and inoculation

In this experiment, Rice blast fungus *M*. *oryzae* strain ZD1, the predominant strain in Northern China, was utilized. The strain was cultivated on half-strength Murashige & Skoog (1/2 MS) solid medium, with a 16 h light / 8 h dark photoperiod. For panicle blast resistance recognition, a 2 mL spore suspension at a concentration of 1×10^5^/mL was used to inject each panicle at the booting stage of rice. In total, fifty panicles of each cultivar were injected, with three replicates measured. Panicle blast resistance was measured using the 0–9 scale of the Standard Evaluation System for Rice (International Rice Research Institute, 2013).

### Sampling and phenotyping

Samples were collected and recorded at 4, 7, 11, 15, and 25 days after inoculation (DAI), each including five replicates. Two leaves were collected from three plants in infected and non-infected resistant and susceptible cultivar groups per treatment at each time point, placed in liquid nitrogen, and stored at -80°C.

### Phenylpropanoid pathway enzyme activity determination

For assessment of PAL and PPO activity, 100 mg leaf tissues were homogenized with 6 mL of ice-cold borate buffer (5 mM, pH 8.8) using a pre-chilled mortar and pestle. The resulting homogeneity was then centrifuged at 8,000 g for 20 min at 4°C. The supernatant was mixed with 0.02 M phenylalanine and distilled water and used as crude extract. After incubating at 30°C for 30 min, PAL activity in the extract were measured at a wavelength of 490 nm. And PPO activity was assessed following oxidation of catechol at 420 nm.

### ROS scavenging enzyme activity determination

For determination of ROS scavenging enzyme activity, leaf samples (100 mg) were homogenized in ice-cold buffer (pH 7.8) with 10 mL of 25 mM potassium phosphate containing 0.2 mM EDTA and 2% polyvinylpyrrolidone. Then, the resulting homogeneity was then centrifuged at 10,000 g for 25 min at 4°C, and the supernatant was used as enzyme extract to determine the activity of ROS scavenging enzymes (SOD, POD and CAT). All spectrophotometry analyses were then conducted on an SP-756P spectrophotometer (Shanghai Spectrum Instruments Co., Ltd, Shanghai, China). SOD activity was measured by a SOD-1-W detection kit (Suzhou Comin Biotechnology Co. Ltd., Suzhou, China). POD activity was assayed using a POD-1-Y detection kit (Suzhou Comin Biotechnology Co. Ltd.), while CAT activity was determined using a CAT-1-Y detection kit (Suzhou Comin Biotechnology Co. Ltd.) by measuring H_2_O_2_ decomposition as the decrease in absorbance at 240 nm.

### Detection of H_2_O_2_

Each leaf samples (100 mg) were collected and assessed the H_2_O_2_ accumulation using H_2_O_2_ detection kit (Beijing Applygen Technologies Inc.) at a wavelength of 415 nm.

### Untargeted metabolomic profiling

Rice tissue were harvested and flash-frozen in liquid N_2_ immediately after collection. Samples were lyophilized and then stored at -80°C. The extraction solvent containing isopropanol/acetonitrile/water (3:3:2, v/v/v) was added to each freeze-dried sample, which using 10 g/mL umbelliferone as an internal standard. After sonicated for 1 h, the mixture was centrifuged at 14,000 rpm for 10 min at 4°C. The collected supernatant was used for liquid chromatography-mass spectrometry (LC-MS) analysis. The LC-MS system employed was an Agilent UHPLC-Q Exactive HF-X 1290 instrument coupled to an Agilent 6540 ultra-high-definition quadrupole time-of-flight mass spectrometer with an electrospray ionization source. Separation of components was achieved by use of an ACQUITY rapid resolution high definition (RRHD) UPLC HSS T3 column (1.8 μm, 2.1 100 mm), with mobile phases A (95% water/5% acetonitrile plus 0.1% formic acid), and B (5% water/95% acetonitrile plus 0.1% formic acid). The gradient for separation was 1% mobile phase B for 2 min, 35% for 3 min, 60% for 8 min, 70% for 10 min, 83% for 20 min, 90% for 22 min, 100% for 26 min, and then again for 28 min. The flow rate was 0.3 mL/min and 5 μL liquors of sample were loaded for each individual analysis. The capillary voltage and spray shield were set to 3500V and the sheath gas and nebulizer gas were set to 10 L/min and 12 L/min, respectively, both at a temperature of 350°C. Spectra were acquired between m/z 50 and 1200, with collision energy in MS/MS mode set to 20V. All chemicals used in the experiment, such as LC-MS-grade acetonitrile, isopropanol, distilled water and formic acid, were of the highest purity, purchased from Fisher Scientific (Waltham, MA, USA), and standards were obtained from Sigma-Aldrich (Beijing, China).

### Data analysis

In this study, all data are presented as the mean ± SE of three biological replicates. The significance of differences was analyzed by using a one-way analysis of variance (ANOVA) with Duncan’s multiple range test at a 5% significance level.

Metabolomics data was pre-processed with ProgenesisQI software (Waters Corp., Milford, USA). LC-MS analysis was conducted with m/z values from ionization products in positive and negative ion modes and matched with METLIN database (http://metlin.scripps.edu/), HMDB database (http://www.hmdb.ca/) and KEGG Compound database (https://www.kegg.jp/kegg/compound/). Principal Component Analysis (PCA) and Orthogonal Partial Least Squares Discriminant Analysis (OPLS-DA) were performed with R package ropls. Differentially accumulated metabolites (DAMs) were assessed with the KEGG database (http://www.kegg.jp/). Significance was determined by assessing Variable Importance in Projection (VIP) values and conducting One-Way Analysis of Variance (ANOVA) or Two-tailed t-tests with Student’s t-test *p-values* less than 0.05, with VIP values that exceed 1.0.

## Results

### Dynamic changes in phenotypes of resistant and susceptible rice cultivars following *M*. *oryzae* panicle infection

**Fig 1:**
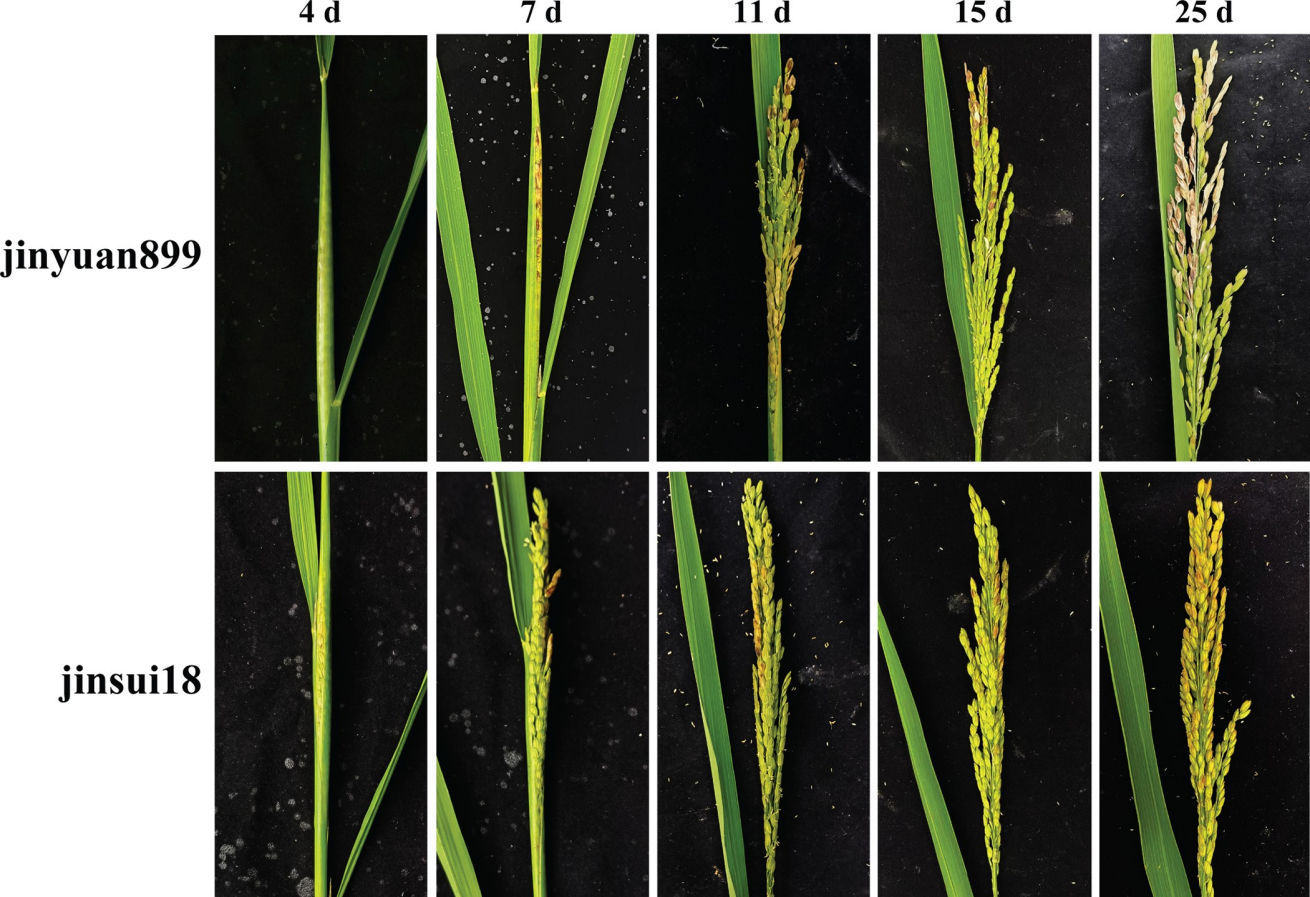
Photographs of resistant and susceptible cultivars against *M*. *oryzae*. Phenotype changes in resistant cultivar jinsui18 and susceptible cultivar jinyuan899 from 4–25 days after inoculation are shown.

Resistant (jinsui18) and susceptible (jinyuan899) cultivars were simultaneously inoculated in panicles with rice blast fungus *M*. *oryzae*. We then observed and compared phenotypic changes between resistant and susceptible cultivars from 4 to 25 DAI, and the results are shown by Fig 1. From DAI4−DAI11, there were no obvious phenotypic differences between resistant and susceptible cultivars. There were no phenotypic changes in their appearance compared with plants inoculated at DAI4. Panicles appeared slightly broken but not pulled out, and leaf sheaths and panicles had turned slightly brown at DAI7. Some grains had turned brown, and some panicles were pulled out at DAI11. However, from DAI15 to DAI25, the performance of resistant and susceptible cultivars differed markedly. Grains of the resistant cultivar became browner, and some disappeared, while grains of the susceptible cultivar dried up, and there were obvious disease spots on branches.

**Fig 2:**
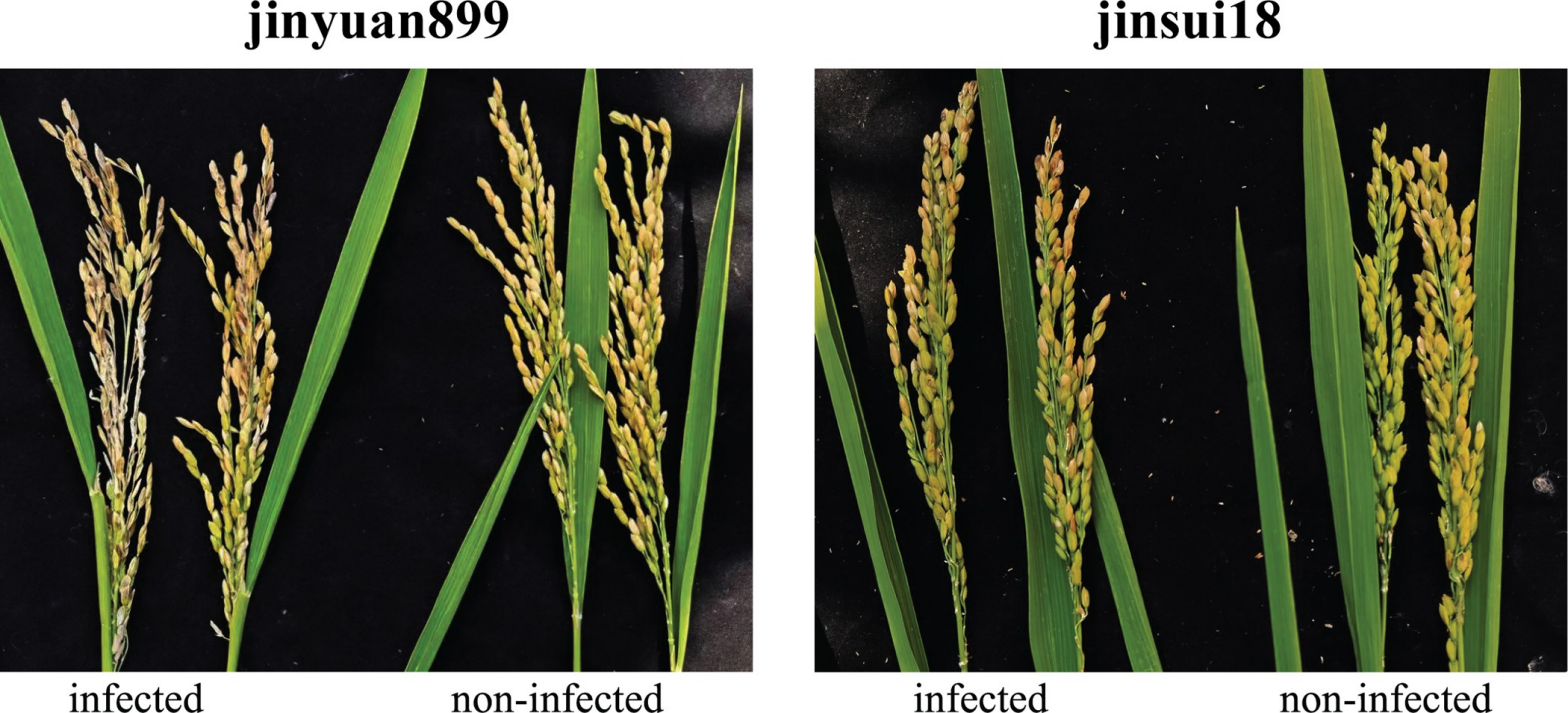
Photographs of resistant and susceptible cultivars under infected and non-infected conditions at 40 days after inoculation.

Panicle blast resistance was investigated in mature rice which at 40 days after inoculation, and the performance of both cultivars is shown in Fig 2. There were significant differences in resistance between resistant and susceptible cultivars (Table 1).

**Table 1:** Results for resistant and susceptible rice cultivars infected with *M*. *oryzae*.

Samples		Number of infected spikelets	Incidence rate (%)	Loss rate (%)	Incidence level	Resistance
**jinyuan899**	I	50	100	49.5	7	S
	II	50	100	49.3	7	S
	III	50	100	50.0	7	S
	average	50	100	49.6	7	S
**jinsui18**	I	50	32.5	1.6	1	R
	II	50	37.5	1.9	1	R
	III	50	39.0	2.0	1	R
	average	50	36.3	1.8	1	R

### Dynamic changes in defense enzyme activities and H_2_O_2_ accumulation in resistant and susceptible rice cultivars following *M*. *oryzae* infection

**Fig 3:**
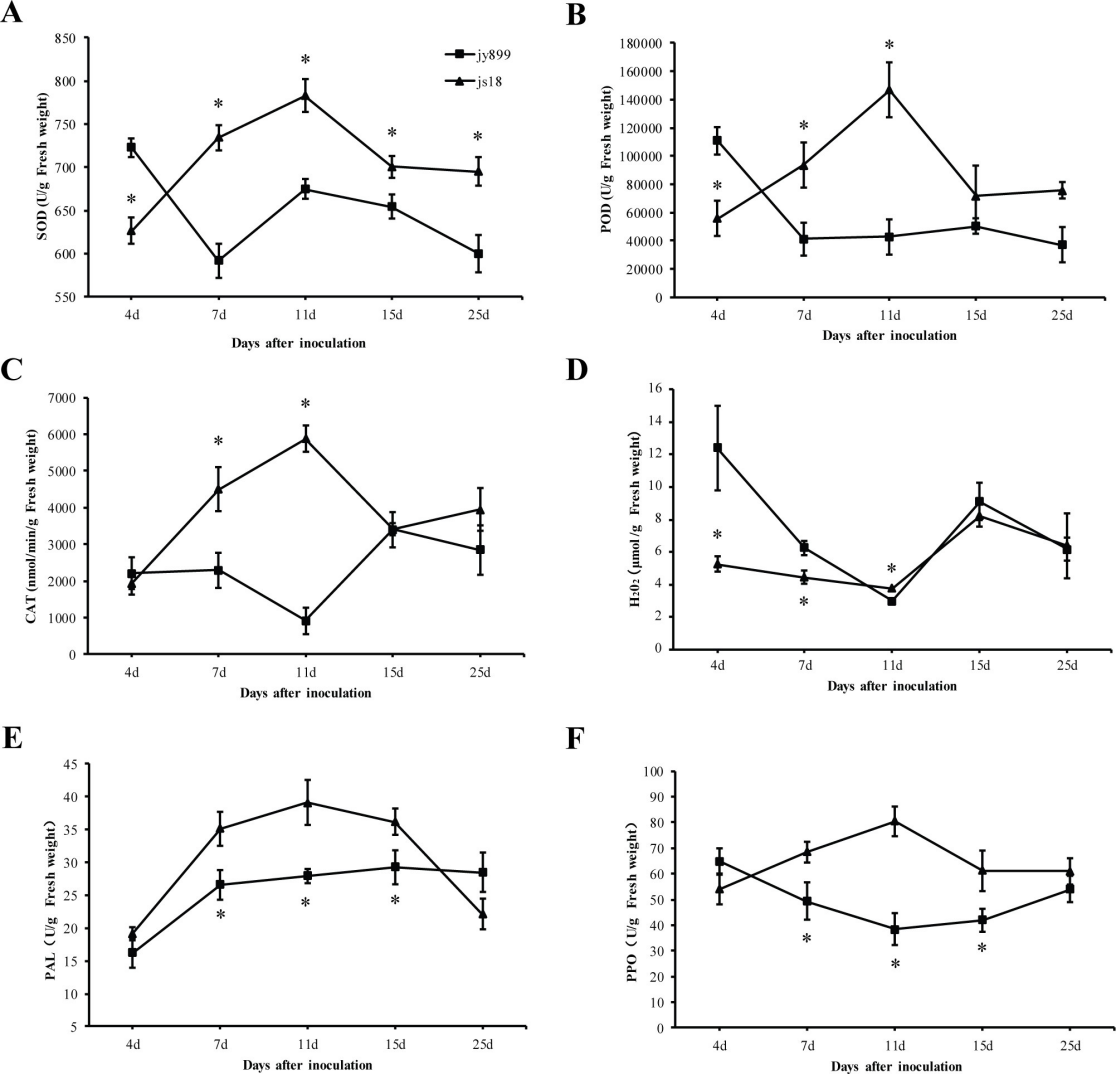
Dynamic changes in the activities of defense enzymes SOD (A), POD (B), CAT (C), PAL (E), PPO (F) and H_2_O_2_ accumulation (D) for resistant and susceptible cultivars at 4–25 DAI with *M*. *oryzae*. (*p <0.*05*).

We measured changes in the activities of five defense enzymes in rice leaves, namely SOD, PPO, CAT, POD, and PAL, related to plant resistance from DAI4 to DAI25. The differences in the activities of these enzymes between the resistant and the susceptible cultivars were evident (Fig 3). For the resistant cultivar jinsui18, SOD, PPO, and CAT activities increased from DAI4 to their peak at DAI11, after which the activities decreased. On the other hand, the susceptible cultivar jinyuan899 had the opposite trend, with its activities first decreasing to the minimum at DAI7, then gradually increasing (Fig 3A–3C). The content of H_2_O_2_ gradually decreased DAI4 to their low peak at DAI11, and then gradually increasing in both resistant and susceptible cultivar. However, the rate of reduction in H_2_O_2_ content was greater in susceptible varieties from DAI4 to DAI11 (Fig 3D).

Moreover, PPO activity increased in the resistant cultivar until it reached its peak at DAI11, while in the susceptible cultivar it decreased to its lowest value at DAI7 and was then maintained at a low level (Fig 3E). PAL activity in both the resistant and the susceptible cultivars increased continuously after inoculation, but the rate of increase was significantly higher in the resistant cultivar. PAL activity reached its peak in the resistant cultivar at DAI11, and then decreased. In the susceptible cultivar, it reached the peak at DAI15, and then slowly decreased (Fig 3F). Furthermore, the differences in the activities of PPO, CAT, POD and PAL between the resistant and susceptible cultivars were the largest at DAI11, while SOD activity showed the greatest disparity at DAI7.

### Metabolic profiling by Ultra-performance LC-MS analysis at DAI11

**Fig 4:**
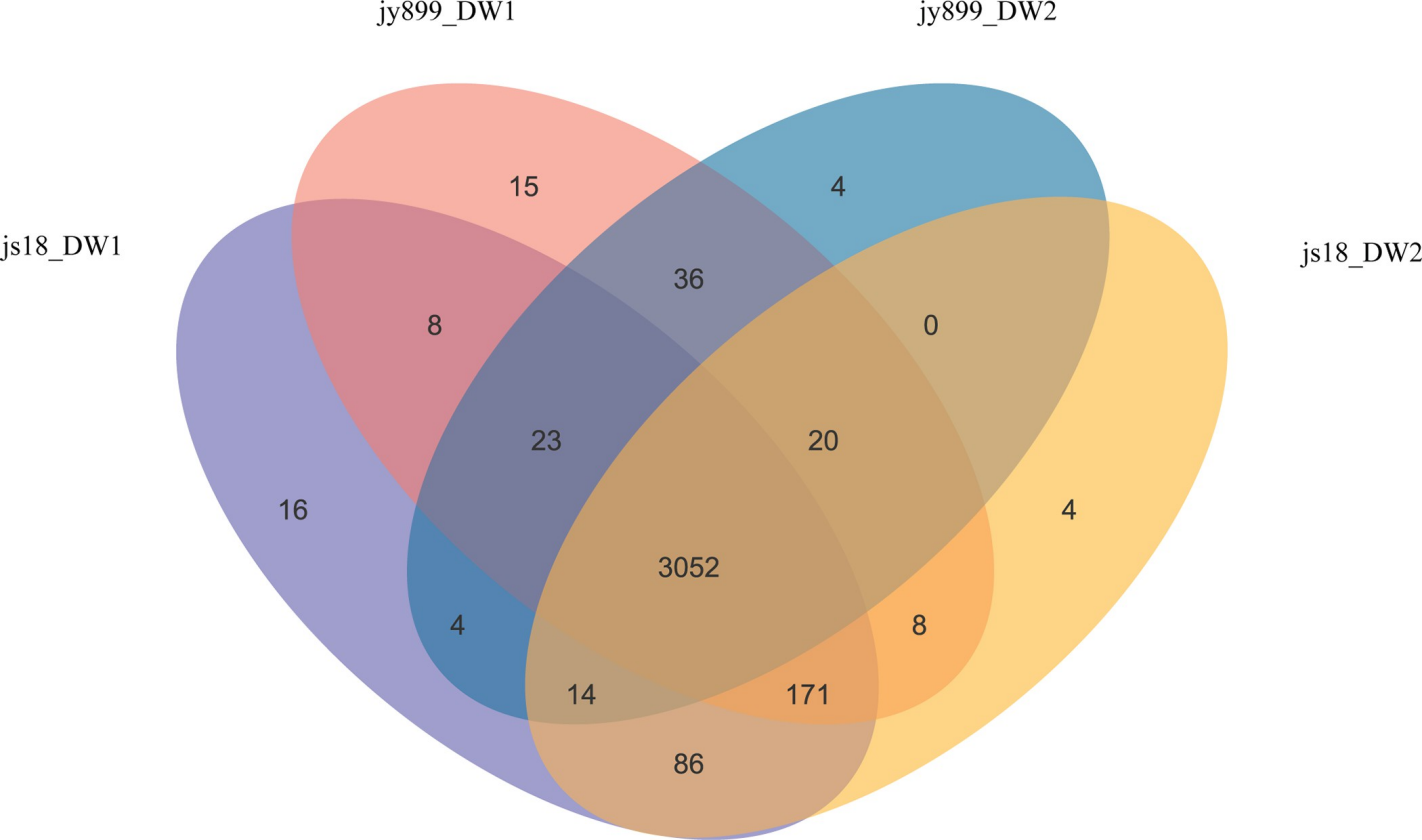
Venn diagram of metabolic profiling in resistant and susceptible varieties. Jy899_DW1 is the metabolome of susceptible cultivar jinyuan 899 infected with *M*. *oryzae* sampled at DAI11. jy899_DW2 is the metabolome of susceptible cultivar jinyuan 899 without infection, sampled at the same time as jy899_DW1. js18_DW1 is the metabolome of resistant cultivar jinsui18 infected with *M*. *oryzae* sampled at DAI11. js18_DW2 is the metabolome of resistant cultivar jinsui18 without infection sampled at the same time as js18_DW2.

Metabolomic analysis was conducted on resistant and susceptible cultivars of rice at DAI11, where the differences in defense enzymes activities were most pronounced. As listed in Table 2, the number of metabolites from each sample was determined. The masses of the analyte peaks were compared with the commercial reference standard compounds, resulting in the identification of 3461 metabolites (Fig 4 and S1 Table). Of these, 3052 metabolites were detected in all four samples. 3453 metabolites were detected in infected samples, 3422 in non-infected control samples and 3300 metabolites were detected in both resistant and susceptible cultivar samples. This data provides valuable evidence regarding the differences between resistant cultivar jinsui18 and susceptible jinyuan899.

**Table 2:** Metabolic profiling results.

Samples	Total metabolites	Descriptions
**jy899_DW1**	3333	Infected jinyuan899 sampled at DAI11
**jy899_DW2**	3153	Non-infected jinyuan899 control sampled at DAI11
**js18_DW1**	3374	Infected jinsui18 sampled at DAI11
**js18_DW2**	3351	Non-infected jinsui18 control sampled at DAI11

### Metabolome differences between resistant and susceptible rice cultivars in infected and non-infected samples

**Fig 5:**
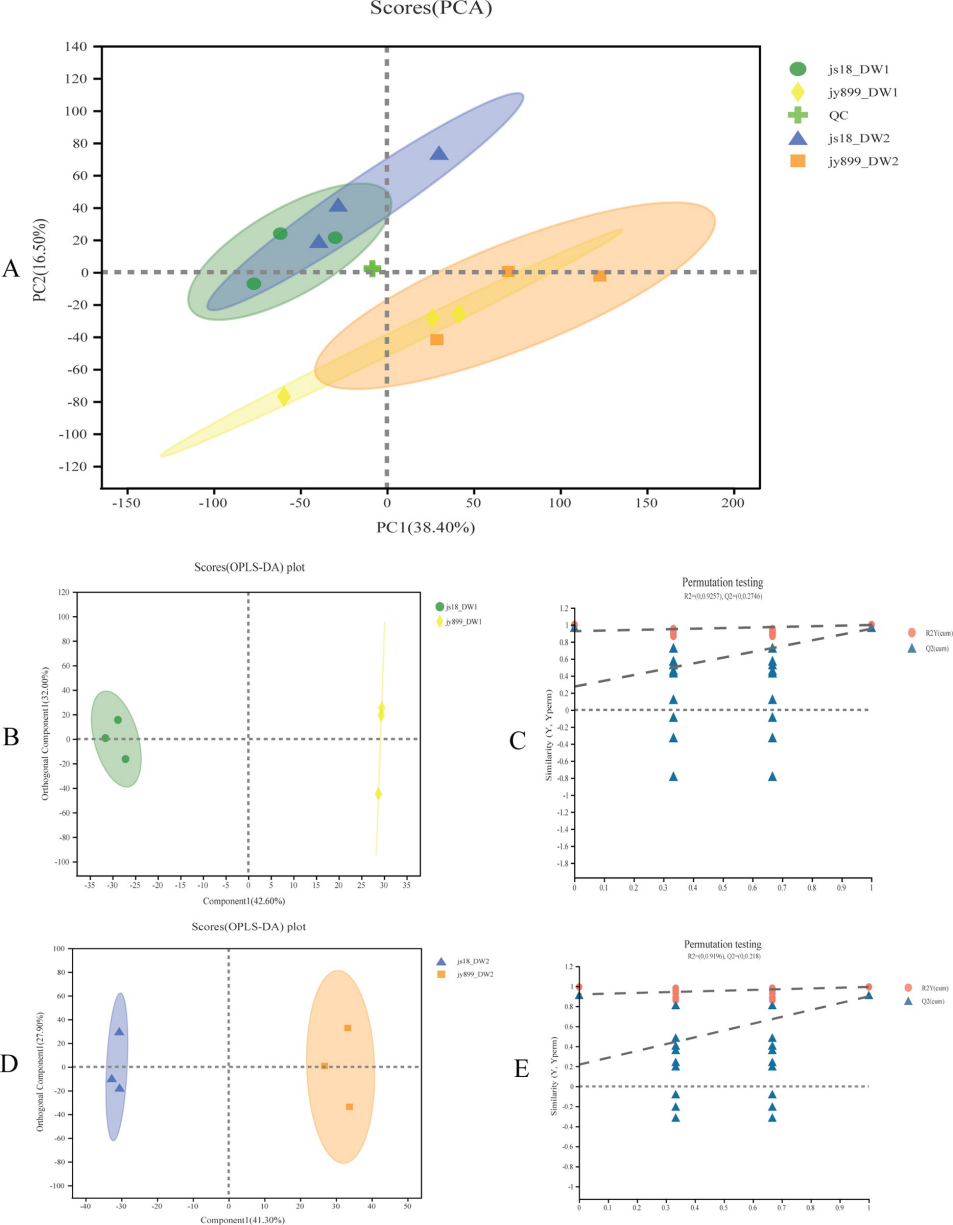
PCA and OPLS-DA of metabolite profiling data. Jy899_DW1 is the metabolome of susceptible cultivar jinyuan899 infected with *M*. *oryzae* sampled at DAI11. jy899_DW2 is the metabolome of susceptible cultivar jinyuan899 without infection, sampled at the same time as jy899_DW1. js18_DW1 is the metabolome of resistant cultivar jinsui18 infected with *M*. *oryzae* sampled at DAI11. js18_DW2 is the metabolome of resistant cultivar jinsui18 without infection sampled at the same time as js18_DW1. (A) PCA scores of metabolomes in rice panicles for infected and non-infected resistant and susceptible cultivars. OPLS-DA scores plots (B) and permutation tests (C) for metabolite profiling of resistant cultivar jinsui18 and susceptible cultivar jinyuan899 with *M*. *oryzae* infection. OPLS-DA scores plots (D) and permutation tests (E) for metabolite profiling of resistant cultivar jinsui18 and susceptible cultivar jinyuan899 without *M*. *oryzae* infection.

Our PCA of metabolites in four samples revealed a distinct separation between resistant and susceptible rice cultivars, with the first two principal components accounting for 54.9% of the total variance of the data. This revealed a larger discrepancy in metabolic activity between resistant and susceptible cultivars when compared to infected and non-infected samples (Fig 5A). The results of PCA indicated that the majority of the variance in the metabolites data originated from the cultivars.

After constructing the OPLS-DA models to investigate the differences between resistant and susceptible cultivars, we observed a distinct clustering of cultivars in both infected and non-infected conditions (Fig 5B–5E). The OPLS-DA models of fungi-challenged panicles at DAI11 demonstrated an impressive variance of 94.6% and 93.2% for the first two components, respectively, compared to 81.9% and 79.8% for infected and non-infected samples, respectively. Furthermore, our permutation tests demonstrated that the OPLS-DA models were valid, with the plots indicating a clear distinction between resistant and susceptible cultivars in both infected and non-infected samples.

**Fig 6:**
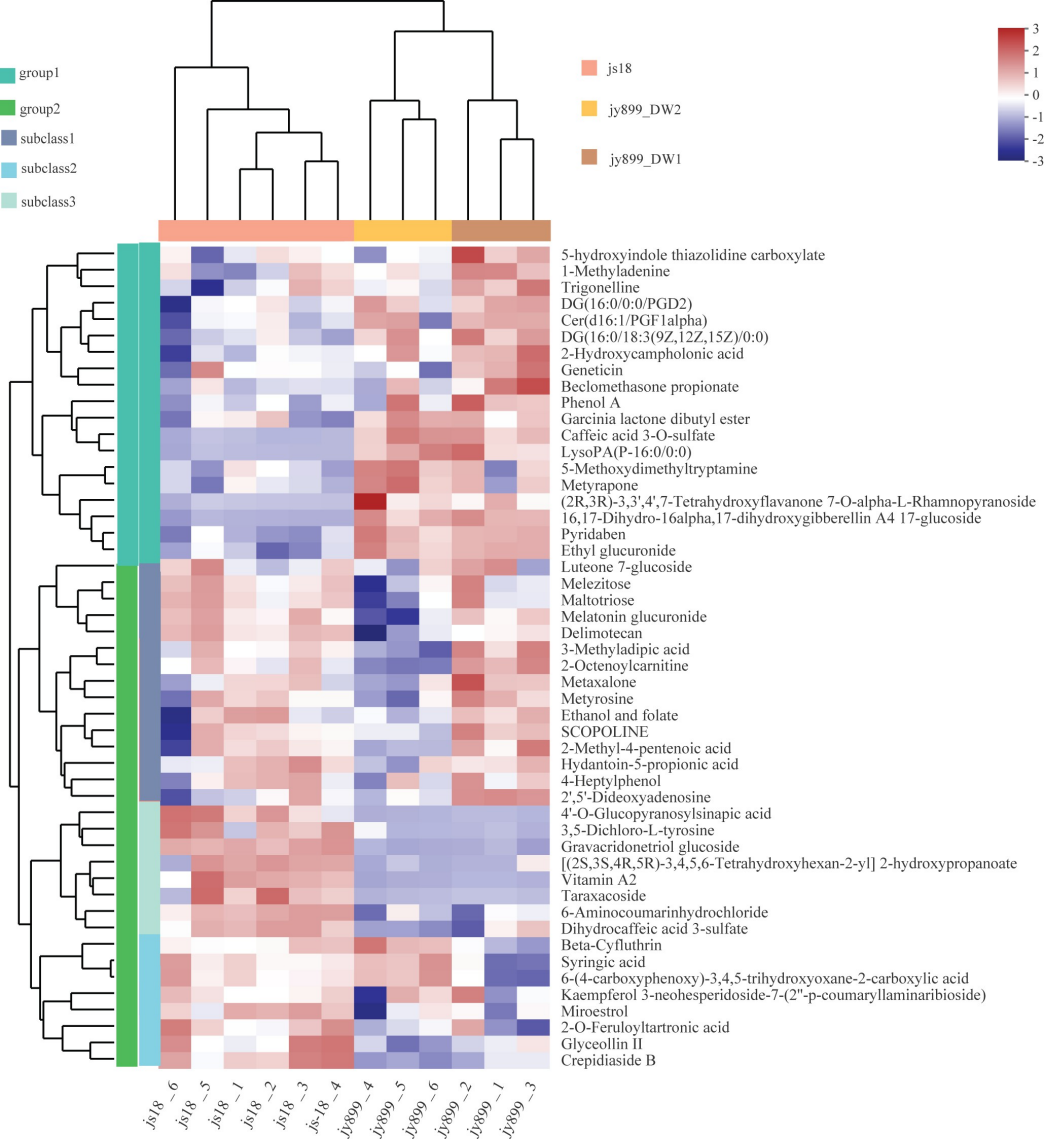
Heatmap analysis of representative differential metabolites in four groups. Jy899_1 and jy899_2, jy899_3 are the metabolome of susceptible cultivar jinyuan899 infected with *M*. *oryzae* sampled at DAI11 with three repeats; jy899_4, jy899_5, and jy899_6 are the metabolome of susceptible cultivar jinyuan 899 without infection sampled at the same time as jy899_1 to jy899_3; js18_1 to js18_3 are the metabolome of resistant cultivar jinsui18 infected with *M*. *oryzae* sampled at DAI11 with three repeats. js18_4, js18_5, and js18_6 are the metabolome of resistant cultivar jinsui 18 without infection sampled at the same time as js18_1 to js18_3. group1. group1, metabolites with low abundance in resistant cultivar jinsui18 and high abundance in susceptible cultivar jinyuan899; group2, metabolites with high abundance in resistant cultivar jinsui18 and low abundance in susceptible cultivar jinyuan899. group2 can be divided into three subtypes (subclass1, subclass2, subclass3) according to patterns in abundance between infected and non-infected samples for susceptible cultivar jinyuan899.

Furthermore, clusters generated by the heatmap analysis of representative differential metabolites in the four samples exhibited obvious segregation between resistant and susceptible cultivars, indicating distinct differences in their accumulated metabolomes (Fig 6). Differential metabolites could be delineated into two groups based on their accumulation patterns: Group 1 metabolites manifested high accumulation in the susceptible cultivars jinyuan899 and low accumulation in the resistant variety jinsui18, whereas Group 2 metabolites presented high levels in the resistant cultivar but low levels in the susceptible cultivar. The Group 2 metabolites could be further divided into three subcategories. Subclass 1 metabolites had high accumulation in infected samples, Subclass 2 metabolites with low indications in infected samples of the susceptible variety, and all other metabolites falling into Subclass 3. Group2 metabolites did not attribute significantly to any differential patterns between infected and non-infected samples in the resistant variety. These findings further corroborate the significant disparities between resistant and susceptible varieties of their metabolomes, with the susceptible cultivar being more prone to changes inflicted by the rice blast fungus.

**Fig 7:**
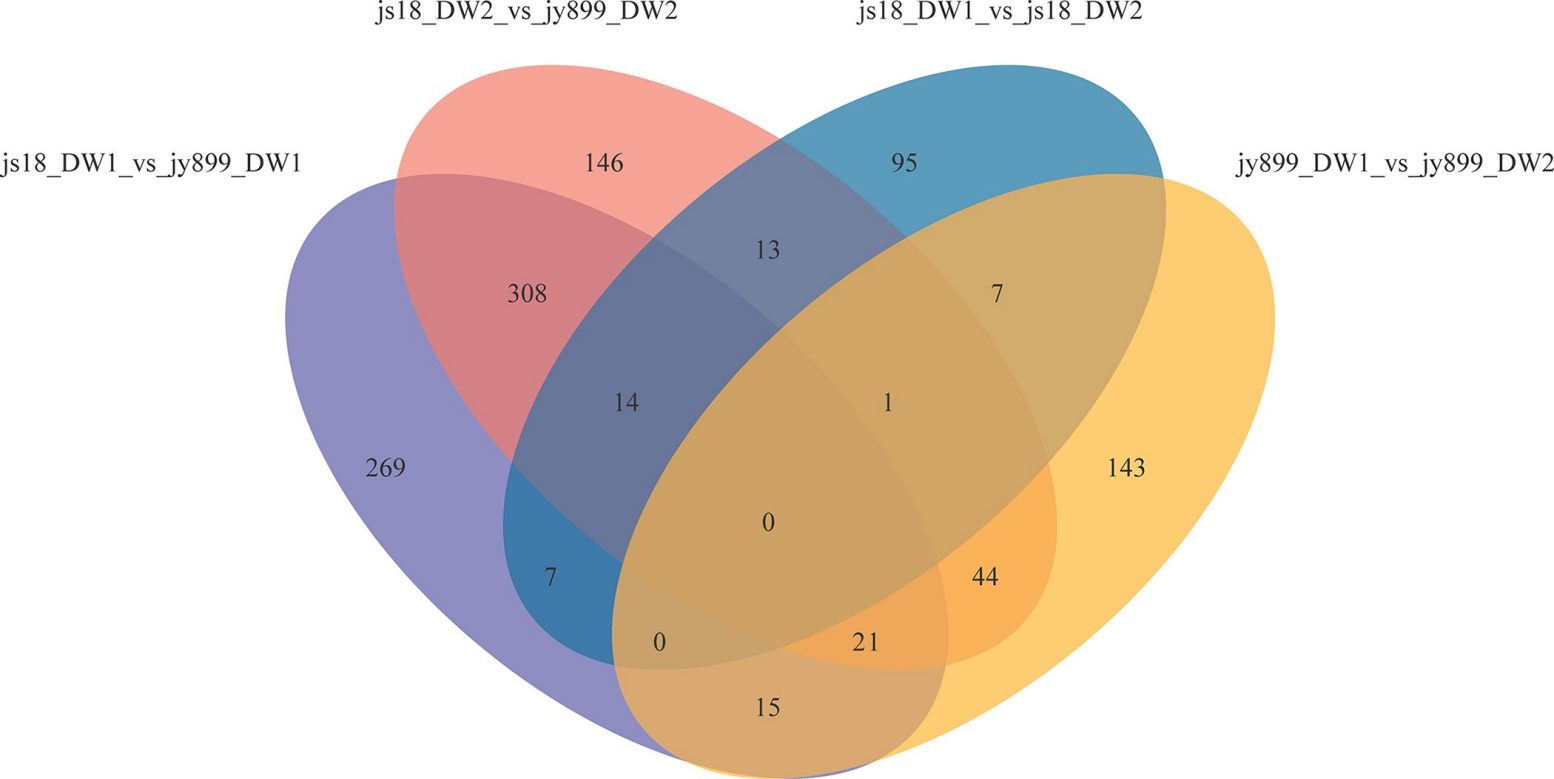
Venn diagram of differential metabolites in four groups. Js18_DW1_vs. jy899_DW1 represents differential metabolites between resistant cultivar jinsui18 and susceptible cultivar jinyuan899 infected with *M*. *oryzae*; js18_DW2_vs. jy899_DW2 represents differential metabolites between resistant cultivar jinsui18 and susceptible cultivar jinyuan899 without infection; js18_DW1_vs. js18_DW2 represents differential metabolites between infected and non-infected samples of resistant cultivar jinsui18; jy899_DW1_vs. jy899_DW2 represents differential metabolites between infected and non-infected samples of susceptible cultivar jinyuan899.

Subsequently, the OPLS-DA analysis of the four sample comparisons revealed 1083 differentially accumulated metabolites (DAMs) with corresponding variable importance in projection (VIP) and Student’s t-test values higher than 1 and 0.05 respectively (Fig 7 and S2 Table). In the js18_DW1 vs. js18_DW2 comparison, which reflected differences in metabolomes between infected and non-infected samples in the resistant cultivar jinsui18, 95 of 137 DAMs were unique in this comparison. In the jy899_DW1 vs. jy899_DW2 comparison, 143 out of 231 DAMs were unique, which reflected differences in metabolomes between infected and non-infected samples in the susceptible cultivar jinyuan899. Eight DAMs were present at high levels in infected samples in both resistant and susceptible cultivars when comparing infected and non-infected samples. The detected DAMs may be involved in the defense system of both susceptible and resistant rice cultivars. These metabolites encompassed a variety of compounds, including lipids/lipid-like molecules (e.g. 2-oxo-4-methylthiobutanoic acid, dihydroroseoside, unshuoside A), organoheterocyclic compounds (limonene-1,2-epoxide), organic oxygen compound (6-hydroxypseudooxynicotine), phenylpropanoid/polyketide compound (dhghab), benzenoid compound (methyl n-acetylanthranilate) and organic acid/derivative compound (leucylleucine) (S2 Table).

**Fig 8:**
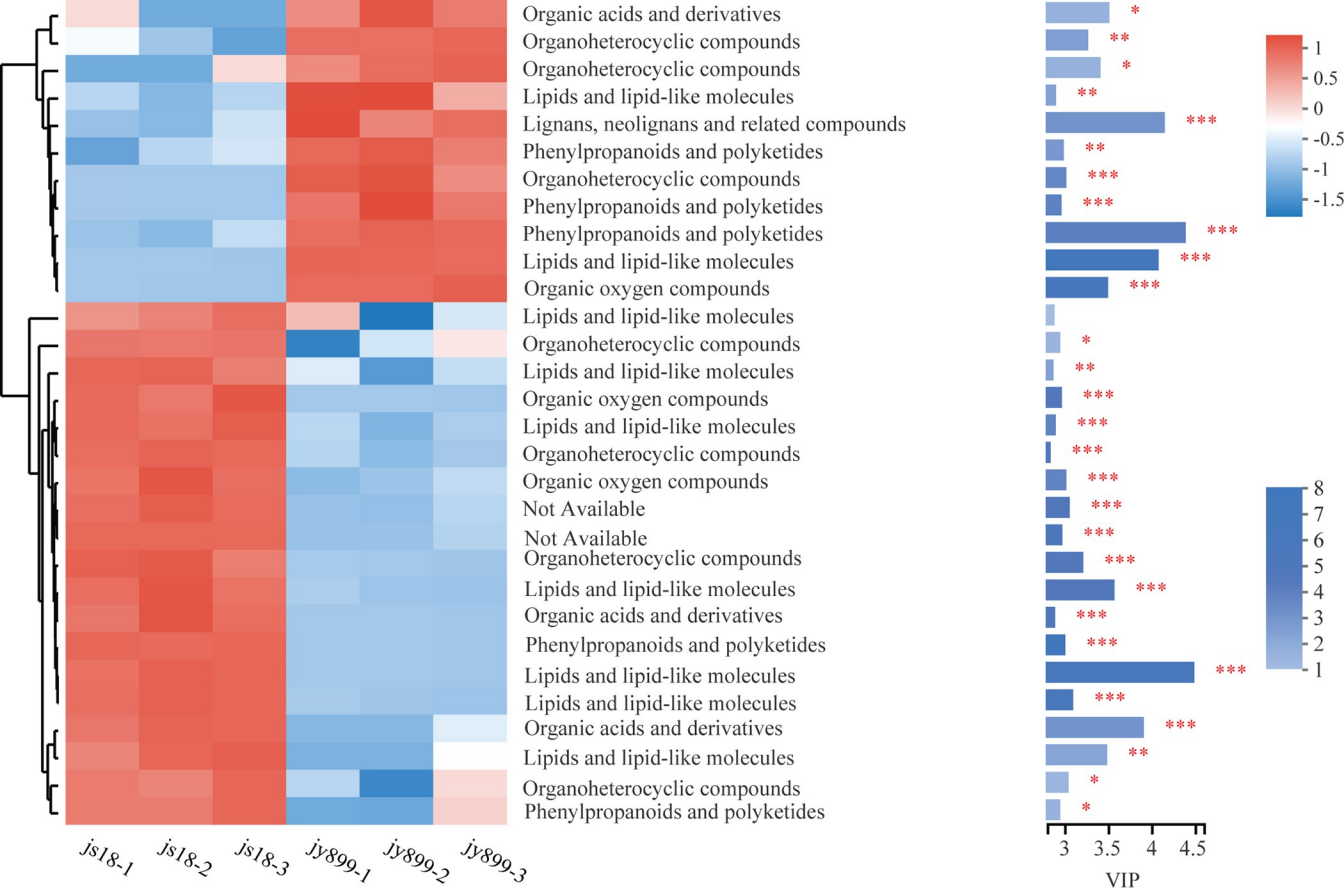
Expression profiling of the top 30 differential metabolites of resistant and susceptible cultivars with *M*. *oryzae* infection. Relative abundances of the top 30 differential metabolites between resistant and susceptible cultivars with *M*. *oryzae* infection. Data are normalized against unit variance. Comparisons were generated via hierarchical cluster analysis using an average linkage method based on Euclidian distance. Shades from blue to red represent increasing metabolite levels (*p <0.*05*, **p *<0*.*01*, ***p <0.*001* for groups compared using one-way ANOVA).

In the js18_DW2 vs. jy899_DW2 comparison without infection at DAI11, 547 DAMs were identified, of which 146 were unique in this comparison. This represents a clear difference in metabolites between jinsui18 and jinyuan899, not related to infection by *M*. *oryzae*. In the js18_DW1 vs. jy899_DW1 comparison, which reflects differences in metabolites between resistant and susceptible cultivars infected by *M*. *oryzae*, we identified 634 DAMs, of which 269 were unique in this comparison; 148 were accumulated at low levels, and 466 were present at high levels in the resistant cultivar (Fig 7 and S3 Table). The top 30 compounds are shown in Fig 8. The major DAMs observed among the studied cultivars were ten lipids/lipid-like molecules, six phenylpropanoids/polyketides, five organoheterocyclic compounds, three organic oxygen compounds, two organic acids/derivatives and one lignan/neolignan and related compound (S2 Table).

### Categorization of DAMs between panicle blast resistant and susceptible cultivars challenged with *M*. *oryzae*

**Fig 9:**
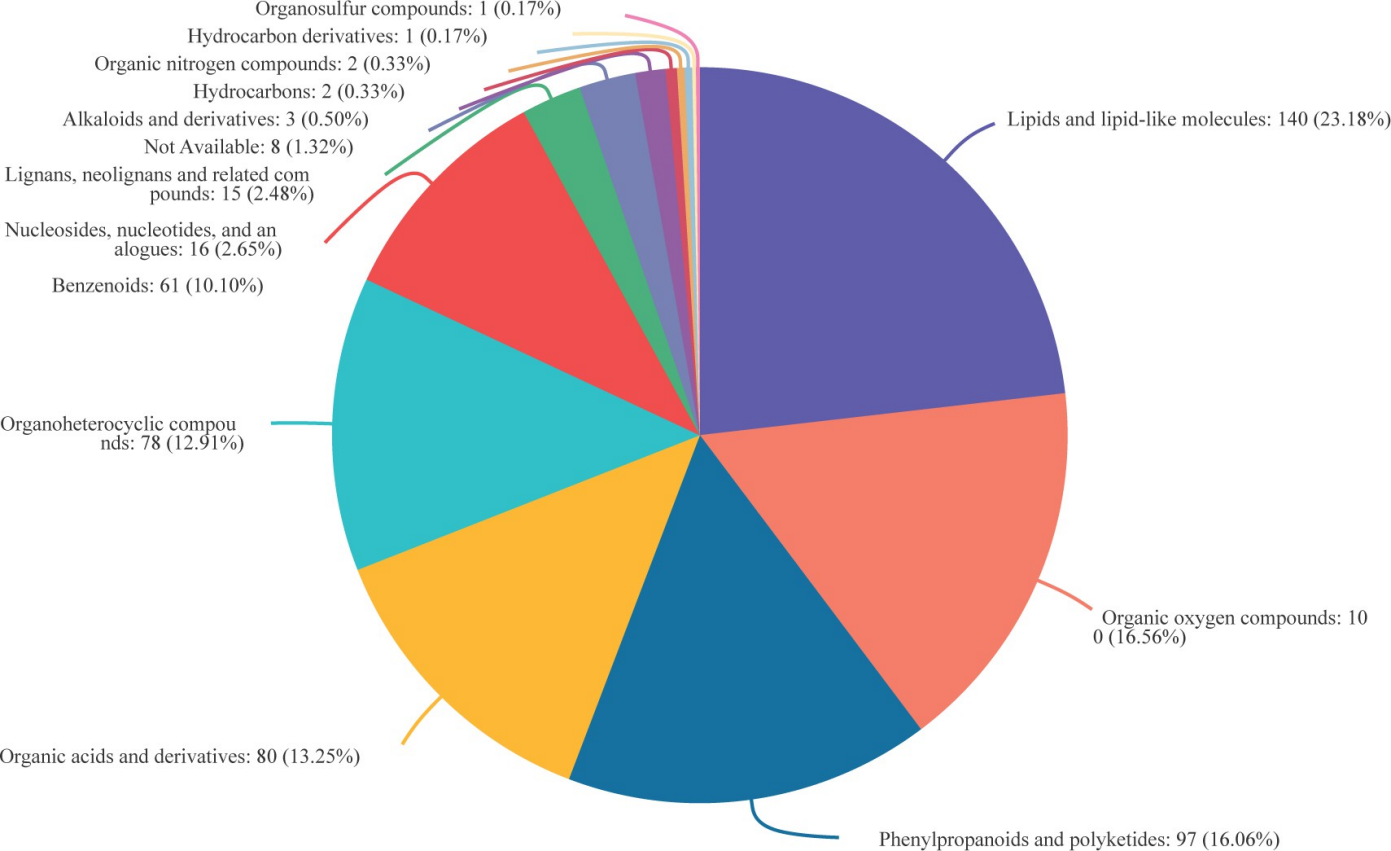
Classification of differential metabolites of resistant and susceptible cultivars with *M*. *oryzae* infection.

In total, 634 DAMs were observed in resistant and susceptible cultivars infected at DAI11, with 604 of these being classified based on super class on the HMDB 4.0 database (Fig 9 and S3 Table). Lipids and lipid-like molecules, such as phenol lipids (66), fatty acyl (45) and steroids/steroid derivatives (20) were the most frequent DAMs, accounting for 23.18% (140). This was followed by organic oxygen compounds (16.56%, 100), phenylpropanoids and polyketides (16.06%, 97), organoheterocyclic compounds (13.25%, 80), organic acids and derivatives (12.91%, 78), benzenoids (10.10%, 61) and few nucleosides, nucleotides and analogues (16), lignans, neolignans and related compounds (15), alkaloids and derivatives (3), and hydrocarbons (2).

### Metabolic pathways of DAMs between panicle blast resistant and susceptible cultivars challenged with *M*. *oryzae*

**Fig 10:**
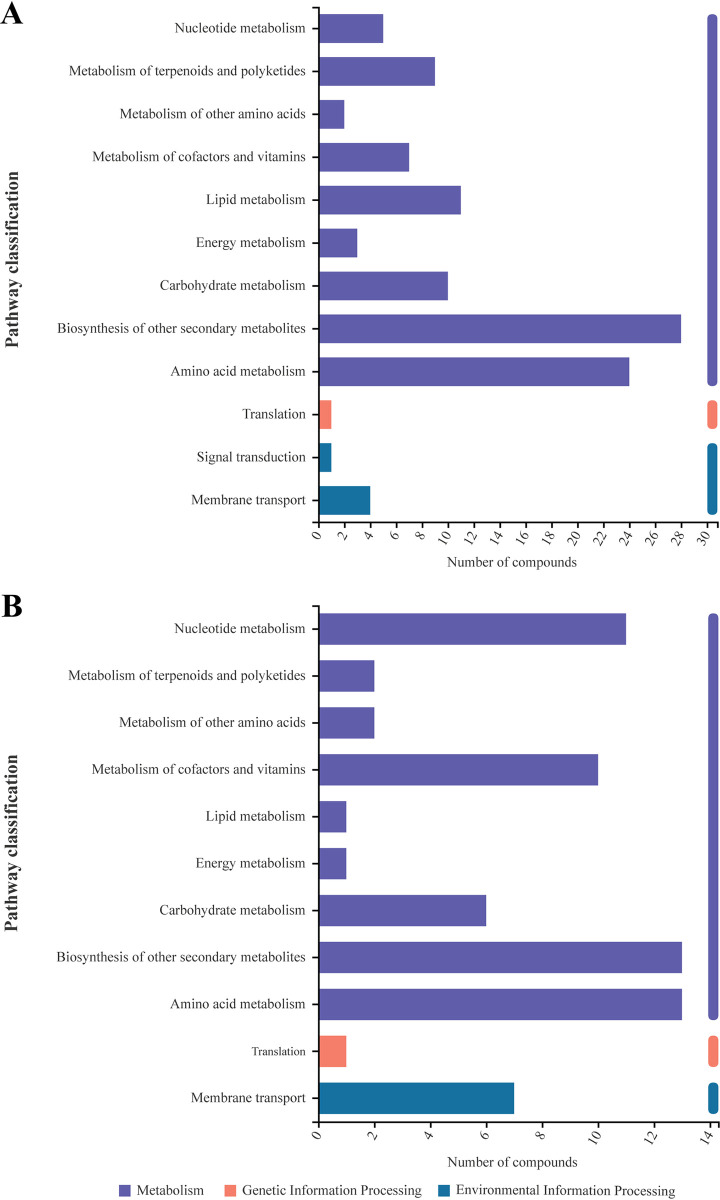
Pathway classification of differential metabolites of resistant and susceptible cultivars with *M*. *oryzae* infection. (A) Pathway classification of differential metabolites between resistant cultivar jinsui18 and susceptible cultivar jinyuan899 with *M*. *oryzae* infection. (B) Pathway classification of differential metabolites between infected and non-infected samples for resistant cultivar jinsui18.

Further analysis of the 634 DAMs related to metabolic pathways showed that multiple metabolic pathways may be responsible for changes in rice cultivars challenged with *M*. *oryzae*. As shown in Fig 10A, metabolic pathways with more than 10 DAMs were biosynthesis of other secondary metabolites (28 DAMs), amino acid metabolism (12 of DAMs), lipid metabolism (11), and carbohydrate metabolism (10) (Fig 10A). These metabolic pathways may be related to resistance to rice panicle blast. Among them, metabolites related to the biosynthesis of other secondary metabolism and amino acid metabolism were also the metabolites showing major differences between infected and non-infected samples in the resistant cultivar jinsui18 (Fig 10B).

**Fig 11:**
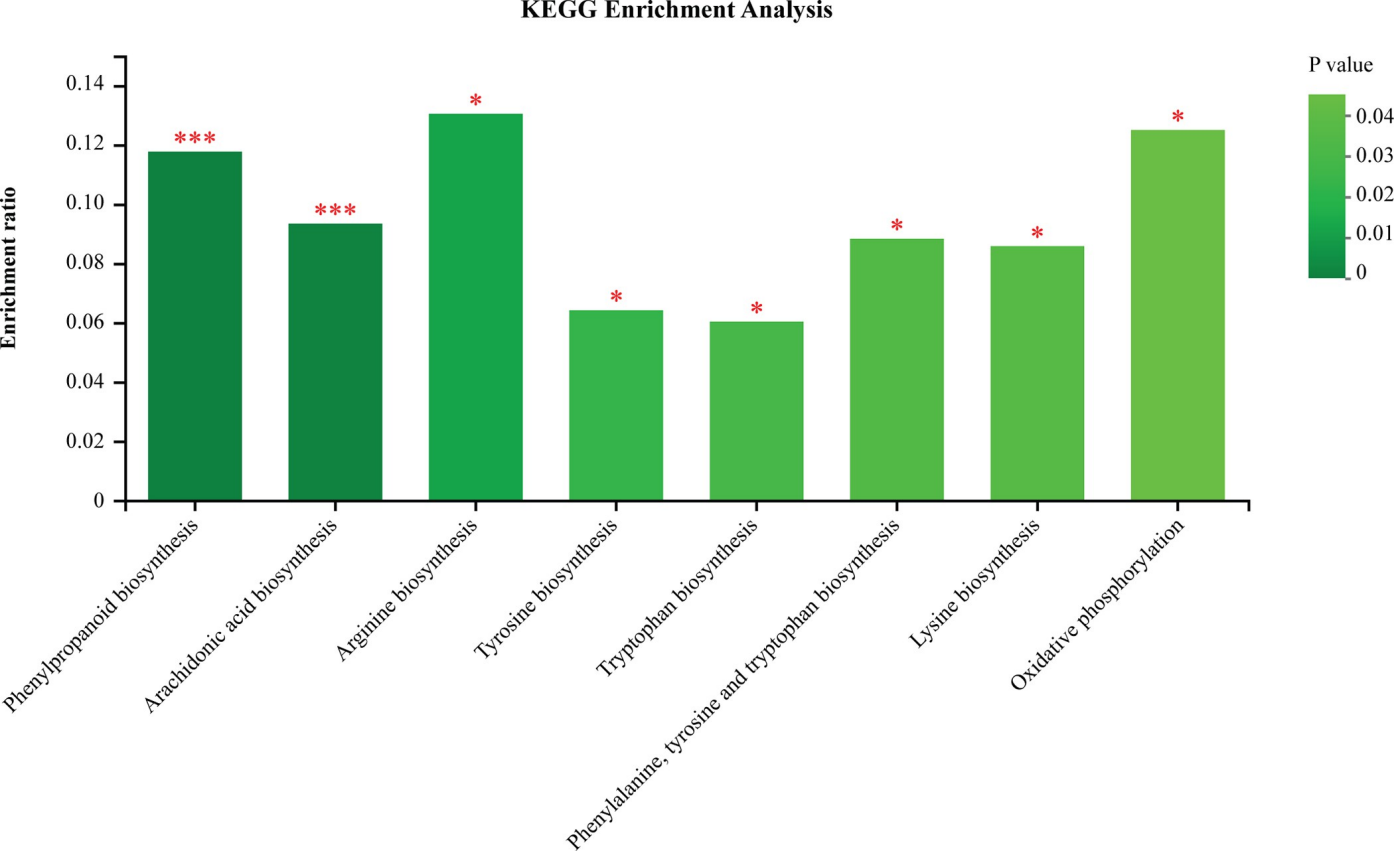
KEGG pathway enrichment of differential metabolites of resistant and susceptible cultivars with *M*. *oryzae* infection (*p <0.*05*, ***p <0.*001*).

KEGG pathway enrichment analysis results indicated that the identified DAMs were enriched in eight metabolic pathways with p-values less than 0.05. These pathways included phenylpropanoid biosynthesis (8 DAMs), arachidonic acid metabolism (7), arginine biosynthesis (3), tyrosine metabolism (5), tryptophan metabolism (6), phenylalanine, tyrosine, and tryptophan biosynthesis (3), lysine biosynthesis (3), and oxidative phosphorylation (1). The results of the analysis showed that each pathway was significantly enriched in DAMs associated with the metabolism of specific metabolites (Fig 11).

## Discussion

### Resistance to rice panicle blast is related to dynamic activities of ROS-scavenging defense enzymes

ROS are involved in many important processes, including signal transduction during pathogen and host plant interactions [24]. ROS production is coupled with ROS degradation by various cellular antioxidants to protect cells from ROS-induced damage to DNA, proteins, and cell membranes, and ultimately cell death [24]. An important part of the antioxidative system is the balance between the generation and scavenging of reactive oxygen species (ROS), which is tightly controlled during plant-*M*. *oryzae* interactions [24]. ROS scavenging defense enzymes are involved in adjusting ROS levels and maintaining resistance to pathogens [25]. El-Komy et al. [26] demonstrated that a resistant faba bean (*Vicia faba*) cultivar had a higher enzymatic ROS scavenging capacity compared to a susceptible one during *Botrytis fabae* infection.Wang et al. [27] reported ROS bursts at 1–3 DAI, and ROS scavenging started at 5 DAI with *M*. *oryzae*. In our current study, we measured the activities of ROS-scavenging defense enzymes at 4–25 DAI, shortly after ROS scavenging began. We demonstrated that panicle blast resistance rice cultivar jinsui18 was far superior at ROS scavenging than susceptible cultivar jinyuan899. The activities of ROS-scavenging defense enzymes (SOD, CAT, POD) increased at first, then decreased in the resistant variety; by contrast, it decreased then increased in the susceptible variety. The activities of ROS-scavenging defense enzymes were higher in the resistant cultivar than in the susceptible cultivar throughout most of the *M*. *oryzae* infection.

We also examined the H_2_O_2_ levels in the resistant varieties after inoculation, and the trend of H2O2 changes in the resistant varieties could be explained by changes in antioxidant enzyme activity, i.e., as the activity of antioxidant enzymes increased, the content of H_2_O_2_ gradually decreased, and as the activity of antioxidant enzymes decreased the content of H_2_O_2_ gradually increased. However, in susceptible varieties, the changes in H_2_O_2_ enzyme content could not be explained by the changes in antioxidant enzyme activity. The changes in H_2_O_2_ content in susceptible varieties may be dominated by *M*. *oryzae*. Fungi also need effective antioxidant measures so that they can better invade the host cells [24].

Therefore, we conclude that resistance to rice panicle blast is related to dynamic activities of ROS-scavenging defense enzymes.

### Resistance to rice panicle blast is related to the phenylpropanoid pathway

Phenylpropanoid pathway defense enzymes are crucial components of plant disease resistance [28–30]. For example, *GmPAL2*.*1* has been identified as a major component of soybean resistance to *Phytophthora sojae* [31]. *OsPAL4* and *OsPAL6* are also important contributors to rice resistance; they could be used as targets in breeding plans to develop enhanced broad-spectrum disease resistance in this crop [12]. In our current study, it was observed that PAL enzyme activity increased in both resistant and susceptible varieties after pathogen inoculation, although this increase was much more pronounced in the resistant one. Thus, PAL enzymes appeared to be involved in protecting against *M*. *oryzae* in panicles in both resistant and susceptible varieties, and PAL activity as higher in the resistant cultivar. Meanwhile, PPO activity first increased, then decreased in the resistant cultivar; by contrast, it decreased then increased in the susceptible variety after pathogen infection. In addition, our KEGG pathway enrichment analysis revealed that DAMs between resistant and susceptible cultivars were enriched in the phenylpropanoid biosynthesis pathway. Therefore, resistance-associated differences in both enzyme activity and defense-associated metabolites (DAMs) related to the phenylpropanoid pathway were observed between the resistant and susceptible cultivars. This indicates that the phenylpropanoid pathway is associated with resistance in rice panicle blast. Further investigation is needed to uncover the underlying molecular and biochemical mechanisms behind this.

### Diverse compounds mediate resistance to rice panicle blast

Previous research has shown that pathogen-induced metabolites are essential contributors to enhanced plant resistance [32]. In this study, 634 distinct defense-associated metabolites were identified between resistant and susceptible cultivars after infection. Organic oxygen compounds, organic acids and derivatives, lipids and lipid-like molecules, phenylpropanoids, and polyketides were among the abundant metabolites. These findings are in line with those of a preceding study characterizing the dominant metabolites in MoSDT1-transgenic rice plants, which demonstrated substantially increased tolerance to *M*. *oryzae* [13].

Organic oxygen compounds and organic acids are key metabolites in infected plants [2]. The accumulation of organic oxygen compounds is often linked to the synthesis of ascorbic acid, an antioxidant known for its role in plant resistance-related properties [33]. Duan et al. [13] reported that organozcbic compounds were uniquely present in *M*. *oryzae*-infected *MoSDT1* transgenic rice plants. Organic acids, a product of incomplete oxidation of photosynthetic products, represent a pool of stored carbon generated during metabolic conversions and enable increased resistance to abiotic and biotic stresses through respiration [34].

One of the main components of biological membranes is lipids, forming an essential boundary between a cell and its external environment [35]. Recent studies point to the critical role of lipids in signal transduction pathways, with the plasma membrane forming an important source for signalling molecules [35]. Lipids are divided into eight categories based on the characteristic hydrophobic and hydrophilic parts of their chemical backbones, namely fatty acyl, glycerolipids, glycerophospholipids, sphingolipids, sterol lipids, phenol lipids, saccharolipids, and polyketides [35]. In the present study, we observed that 66 phenol lipids, 45 fatty acyl, and 20 sterol lipids were the predominant differentially accumulated lipids in panicles of rice cultivars exhibiting either resistance or susceptibility to *M*. *oryzae* infection. However, further investigation is necessary to elucidate the precise mechanisms behind the defence-promoting properties of these metabolites.

Plants contain high levels of conflicting phenylpropanoid molecules that have various physiological activities, such as attraction of pollinators, secondary cell wall growth and defense against plant diseases [36]. The antioxidant property of phenylpropanoids is often attributed to the hydroxyl groups and unsaturated double bonds that react with radical and oxidized ions [36]. Polyketides are one type of phenylpropanoid, known for the range of their chemical structures, which are indispensable for the production of pharmaceuticals. In addition to participating in phenylpropanoid metabolism in plants, some polyketides are capable of producing interesting biological effects [36].

### Multiple metabolic pathways mediate resistance to rice panicle blast

Critical metabolic pathways are activated when plants are infected by pathogens [37]. Therefore, understanding these pathways could provide insight and a specific strategy for strengthening plant resistance systems. Our current study revealed that multiple metabolic pathways with many DAMs between resistant and susceptible rice cultivars maybe responsible for resistance following challenge with *M*. *oryzae*. Analyzing the KEGG pathways uncovered that a multitude of processes within biosynthesis and metabolism, such as biosynthesis of secondary metabolites, amino acid metabolism, lipid metabolism, carbohydrate metabolism, phenylpropanoid biosynthesis, arginine biosynthesis, tyrosine and tryptophan biosynthesis, lysine biosynthesis, arachidonic acid metabolism, tyrosine metabolism, and tryptophan metabolism, are correlated with resistance to panicle blast in rice.

Secondary metabolites produced by plant pathogenic fungi are complex and have unique structures. *M*. *oryzae*, the cause of rice blast disease, is known to produce four types of secondary metabolites, namely melanin, tenuazonic acid, nectriapyrones, and pyriculols [38]. Many of these metabolites are involved in the interaction between *M*. *oryzae* and rice; however, in most cases, the exact metabolites produced in that interaction remain unknown [38]. An interesting example of this is the virulence gene *ACE1*, which is found in *M*. *oryzae* isolates that are specifically recognized by rice cultivars carrying the *Pi33* resistance gene [39]. While it is known that *ACE1* is a secondary metabolism gene, the exact product that is produced has yet to be determined. This highlights the importance of secondary metabolites in the rice-*M*. *oryzae* interaction, and our research aims to uncover additional information in this regard.

Lipid metabolism has an important role in modulating the plant response to biotic and abiotic stresses by altering the structure and signaling of lipids [35,40]. A key mechanism of this process is the hydrolysis of membrane phospholipids by phospholipase D (PLD), which produces the signaling compound phosphatidic acid (PA). PLD-PA is a potentially important signaling complex for regulating lipid metabolism, hormone signaling, and plant defense responses [41]. It has the capability to interact with various proteins and other lipid molecules to modulate action in cytoskeleton dynamics, alter microtubule cytoskeleton, and stimulate hormone and reactive oxygen species (ROS) production [41]. A distinct feature of the PLD-PA complex is that PA can bind to and regulate a broad range of proteins by changing the physical and chemical properties of the lipids [41]. Findings from our study suggest that lipid metabolism was the most significantly changed metabolic process in response to *M*. *oryzae* infection, and it is plausible that a variety of lipid signaling molecules can be produced by alterations in lipid metabolism.

Successful infection by pathogens is heavily contingent on their capacity to utilize available nutrient sources provided by plants, in addition to their ability to penetrate and overpower host plants’ defensive mechanisms [42]. Primary metabolites, particularly certain specialized metabolites, are crucial components of plant development, growth, reproduction processes, in addition to protecting against biotic and abiotic stresses [43,44]. Nutrient metabolism, including amino acid metabolism, arginine biosynthesis, lysine biosynthesis, tyrosine metabolism, tryptophan metabolism, and arachidonic acid metabolism, has been identified to have a substantial impact on the growth, asexuality, differentiation, and virulence of *M*. *oryzae* and other pathogens [6,45–47]. Amino acids participate in the process of nitrogen assimilation and serve as precursors for multiple defense-related secondary metabolites [48]. The gene *MoCPA2* of *M*. *oryzae* encodes a large subunit of arginine-specific carbamoyl phosphate synthase and has been found to be involved in arginine biosynthesis and induction of plant defense responses as well as scavenging ROS during pathogen-plant interactions [6]. The transgenic lines expressing *MoSDT1*, which elicited improved blast resistance in rice, contained significantly more tyrosine and tryptophan, two aromatic amino acids relative to production of a number of defense-related secondary metabolites [49]. Additionally, *MoLys2* in *M*. *oryzae* was determined to be essential for lysine biosynthesis, thus constituting a potential target for development of novel fungicides against *M*. *oryzae* by targeting lysine biosynthesis [50]. Arachidonic acids have been reported to trigger programmed cell death, defense responses in plants, as well as inducing resistance to viruses in potato [51] and tobacco (*Nicotiana tabacum*) [52].

## Conclusions

Our exploration has revealed that dynamic ROS levels and the phenylpropanoid pathway are both associated with resistance to rice panicle blast. Multiple molecules, encompassing organic acids and derivatives, oxygen-containing organic compounds, lipids and lipid-like compounds, as well as phenylpropanoids and polyketides, were reported to be connected to resistance against the disease. In addition, various metabolic pathways such as secondary metabolite biosynthesis, amino acid metabolism, lipid metabolism, phenylpropanoid biosynthesis, arachidonic acid metabolism, arginine biosynthesis, tyrosine metabolism, tryptophan metabolism, phenylalanine, tyrosine, tryptophan biosynthesis, lysine biosynthesis, and oxidative phosphorylation are further linked to resistance to rice panicle blast. Such research outcomes are likely to be beneficial in the optimization of resistance to panicle blast in rice, in addition to providing elementary data relevant to future plant-disease prevention.

## Supporting information

S1 Table Information of 3461 metabolites accumulated between panicle blast resistant and susceptible cultivars challenged with *M*.*oryzae* at DAI11. (XLS)

S2 Table Information of all accumulated metabolites accumulated between panicle blast resistant and susceptible cultivars challenged with *M*.*oryzae* at DAI11. (XLS)

S3 Table Information of 634 differentially accumulated metabolites between panicle blast resistant and susceptible cultivars challenged with *M*.*oryzae* at DAI11. (XLS)
